# Supplementary material for: Global Molecular Response of Paracoccidioides brasiliensis to Zinc Deprivation: Analyses at Transcript, Protein and MicroRNA Levels
Source: J Fungi (Basel). 2023 Feb 21;9(3):281. doi: 10.3390/jof9030281 (PMC10056003; doi:10.3390/jof9030281)
Supplement: Supplementary file 1 [file jof-09-00281-s001.zip › Table S1.pdf]

Table S1. Some RNAs targets of PbZn-miR-1 and PbZn-miR-9.

MicroRNA	Targets	MFE	Functional classification	P-value	
PbZn-miR-1	PADG_02034/C2H2 transcription factor	-22.7 kcal/mol	TRANSCRIPTION	0.049377	3´ UTR
PbZn-miR-1	PADG_02215/ribonuclease P/MRP protein subunit POP8	-24.5 kcal/mol	TRANSCRIPTION	0.023068	3´ UTR
PbZn-miR-1	PADG_02229/Zn(2)-C6 fungal-type domain-containing protein	-25.0 kcal/mol	TRANSCRIPTION	0.018644	3´ UTR
PbZn-miR-1	PADG_02458/DNA-directed RNA polymerase	-25.6 kcal/mol	TRANSCRIPTION	0.014432	3´ UTR
PbZn-miR-1	PADG_04369/splicing factor U2AF 23 kDa subunit	-23.2 kcal/mol	TRANSCRIPTION	0.040012	3´ UTR
PbZn-miR-1	PADG_04404/cactin	-22.7 kcal/mol	TRANSCRIPTION	0.049377	3´ UTR
PbZn-miR-1	PADG_04949/Threonine-tRNA ligase	-24.1 kcal/mol	TRANSCRIPTION	0.027341	3´ UTR
PbZn-miR-1	PADG_05396/ribonuclease H2 subunit C	-24.1 kcal/mol	TRANSCRIPTION	0.027341	3´ UTR
PbZn-miR-1	PADG_05770/general transcription factor 3C polypeptide 5	-22.7 kcal/mol	TRANSCRIPTION	0.049377	3´ UTR
PbZn-miR-1	PADG_07755/Mediator of RNA polymerase II transcription subunit 11	-26.8 kcal/mol	TRANSCRIPTION	0.008636	3´ UTR
PbZn-miR-1	PADG_07959/SAGA-associated factor 11	-24.9 kcal/mol	TRANSCRIPTION	0.019456	3´ UTR
PbZn-miR-1	PADG_02228/ C2H2 transcription factor (AmdA)	-25.0 kcal/mol	TRANSCRIPTION	0.018644	5´ UTR
PbZn-miR-1	PADG_07602/ Suppressor of SUA7 protein 2 homolog	-25.0 kcal/mol	TRANSCRIPTION	0.018644	5´ UTR
PbZn-miR-9	PADG_00069/DNA-binding SARP family transcriptional activator	-23.6 kcal/mol	TRANSCRIPTION	0.033793	3´ UTR
PbZn-miR-9	PADG_00110/HLH transcription factor	-23.4 kcal/mol	TRANSCRIPTION	0.036774	3´ UTR
PbZn-miR-9	PADG_00272/rRNA-processing protein FCF1	-23.6 kcal/mol	TRANSCRIPTION	0.033793	3´ UTR
PbZn-miR-9	PADG_00758/fungal specific transcription factor domain-containing protein	-22.8 kcal/mol	TRANSCRIPTION	0.047347	3´ UTR
PbZn-miR-9	PADG_00876/5'-3' exoribonuclease	-23.1 kcal/mol	TRANSCRIPTION	0.041734	3´ UTR
PbZn-miR-9	PADG_01354/C2H2 transcription factor (RfeC)	-23.0 kcal/mol	TRANSCRIPTION	0.042983	3´ UTR
PbZn-miR-9	PADG_02002/Homeobox domain-containing protein	-23.0 kcal/mol	TRANSCRIPTION	0.043529	3´ UTR
PbZn-miR-9	PADG_02034/C2H2 transcription factor (AmdA)	-24.7 kcal/mol	TRANSCRIPTION	0.020917	3´ UTR
PbZn-miR-9	PADG_02116/pre-rRNA-processing protein ipi1	-23.4 kcal/mol	TRANSCRIPTION	0.037400	3´ UTR
PbZn-miR-9	PADG_02917/CCAAT-box-binding transcription factor	-23.0 kcal/mol	TRANSCRIPTION	0.043529	3´ UTR
PbZn-miR-9	PADG_03453/RNA helicase	-23.1 kcal/mol	TRANSCRIPTION	0.041734	3´ UTR
PbZn-miR-9	PADG_03818/JmjC domain-containing histone demethylation protein	-23.4 kcal/mol	TRANSCRIPTION	0.036774	3´ UTR
PbZn-miR-9	PADG_03842/Mediator of RNA polymerase II transcription subunit 14	-24.4 kcal/mol	TRANSCRIPTION	0.024070	3´ UTR

PbZn-miR-9	PADG_04340/DNA-directed RNA polymerase subunit beta	-23.3 kcal/mol	TRANSCRIPTION	0.038359	3′ UTR
PbZn-miR-9	PADG_04546/rRNA processing protein gar2	-22.9 kcal/mol	TRANSCRIPTION	0.045399	3′ UTR
PbZn-miR-9	PADG_04796/Pre-mRNA-splicing factor rse1	-24.1 kcal/mol	TRANSCRIPTION	0.027341	3′ UTR
PbZn-miR-9	PADG_05585/Pre-mRNA-splicing factor syf1	-25.0 kcal/mol	TRANSCRIPTION	0.018644	3′ UTR
PbZn-miR-9	PADG_05587/U2 small nuclear ribonucleoprotein B	-23.5 kcal/mol	TRANSCRIPTION	0.035252	3′ UTR
PbZn-miR-9	PADG_05787/ATP-dependent RNA helicase FAL1	-22.9 kcal/mol	TRANSCRIPTION	0.045399	3′ UTR
PbZn-miR-9	PADG_06038/COP9 signalosome complex subunit 2	-24.9 kcal/mol	TRANSCRIPTION	0.019456	3′ UTR
PbZn-miR-9	PADG_06607/BZIP family transcription factor	-25.7 kcal/mol	TRANSCRIPTION	0.013594	3′ UTR
PbZn-miR-9	PADG_07203/DNA-directed RNA polymerase III subunit RPC2	-22.9 kcal/mol	TRANSCRIPTION	0.045399	3′ UTR
PbZn-miR-9	PADG_07987/RING finger protein	-22.9 kcal/mol	TRANSCRIPTION	0.045399	3′ UTR
PbZn-miR-9	PADG_07993/pre-mRNA splicing factor	-26.4 kcal/mol	TRANSCRIPTION	0.010250	3′ UTR
PbZn-miR-9	PADG_08146/C2H2 transcription factor	-22.9 kcal/mol	TRANSCRIPTION	0.045399	3′ UTR
PbZn-miR-9	PADG_08283/SAP domain-containing protein	-26.7 kcal/mol	TRANSCRIPTION	0.009014	3′ UTR
PbZn-miR-9	PADG_12215/transcriptional regulator Medusa	-24.1 kcal/mol	TRANSCRIPTION	0.026995	3′ UTR
PbZn-miR-9	PADG_12222/U3 small nucleolar RNA-associated protein	-23.1 kcal/mol	TRANSCRIPTION	0.041734	3′ UTR
PbZn-miR-9	PADG_12326/transcription factor HAP3	-25.3 kcal/mol	TRANSCRIPTION	0.016404	3′ UTR
PbZn-miR-9	PADG_12476/Nucleoside triphosphate pyrophosphohydrolase	-24.8 kcal/mol	TRANSCRIPTION	0.020302	3′ UTR
PbZn-miR-9	PADG_00491/ TATA-binding protein-associated factor MOT1	-26.0 kcal/mol	TRANSCRIPTION	0.012164	5′ UTR
PbZn-miR-9	PADG_00508/ Mediator of RNA polymerase II transcription subunit 10	-23.0 kcal/mol	TRANSCRIPTION	0.043529	5′ UTR
PbZn-miR-9	PADG_00533/ U2-associated protein	-24.2 kcal/mol	TRANSCRIPTION	0.026205	5′ UTR
PbZn-miR-9	PADG_00687/ transcription initiation factor TFIIB	-26.0 kcal/mol	TRANSCRIPTION	0.012164	5′ UTR
PbZn-miR-9	PADG_00848/ DNA-directed RNA polymerases I, II, and III subunit RPABC1	-23.3 kcal/mol	TRANSCRIPTION	0.038359	5′ UTR
PbZn-miR-9	PADG_00861/U4/U6 small nuclear ribonucleoprotein PRP3	-23.9 kcal/mol	TRANSCRIPTION	0.029761	5′ UTR
PbZn-miR-9	PADG_00897/ Homocitrate synthase	-25.8 kcal/mol	TRANSCRIPTION	0.013193	5′ UTR
PbZn-miR-9	PADG_01054/ C6 zinc finger domain-containing protein	-23.1 kcal/mol	TRANSCRIPTION	0.041734	5′ UTR
PbZn-miR-9	PADG_01104/ DNA-directed RNA polymerase III RPC4	-26.3 kcal/mol	TRANSCRIPTION	0.010698	5′ UTR
PbZn-miR-9	PADG_01105/ C6 finger domain-containing protein	-23.1 kcal/mol	TRANSCRIPTION	0.041734	5′ UTR
PbZn-miR-9	PADG_01151/ DNA-directed RNA polymerase II 13.3 kDa polypeptide	-24.2 kcal/mol	TRANSCRIPTION	0.026205	5′ UTR
PbZn-miR-9	PADG_01170/ C2H2 zinc finger domain-containing protein	-23.5 kcal/mol	TRANSCRIPTION	0.035252	5′ UTR

PbZn-miR-9	PADG_01437/ tRNA-splicing endonuclease subunit Sen15	-22.8 kcal/mol	TRANSCRIPTION	0.047347	5´ UTR
PbZn-miR-9	PADG_01442/ protein STB3	-24.5 kcal/mol	TRANSCRIPTION	0.023068	5´ UTR
PbZn-miR-9	PADG_01607/ C6 finger domain-containing protein	-22.7 kcal/mol	TRANSCRIPTION	0.049377	5´ UTR
PbZn-miR-9	PADG_01630/ transcription regulator BDF1	-31.5 kcal/mol	TRANSCRIPTION	0.001147	5´ UTR
PbZn-miR-9	PADG_01691/ Histone acetyltransferase	-22.8 kcal/mol	TRANSCRIPTION	0.047347	5´ UTR
PbZn-miR-9	PADG_01892/ pre-mRNA-processing factor 40	-24.8 kcal/mol	TRANSCRIPTION	0.020302	5´ UTR
PbZn-miR-9	PADG_02185/ C3HC4 zinc finger domain-containing protein	-23.1 kcal/mol	TRANSCRIPTION	0.041734	5´ UTR
PbZn-miR-9	PADG_02308/ pre-mRNA-splicing factor ATP-dependent RNA helicase PRP43	-22.9 kcal/mol	TRANSCRIPTION	0.045399	5´ UTR
PbZn-miR-9	PADG_02356/ RNA polymerase I-specific transcription initiation factor RRN6	-24.8 kcal/mol	TRANSCRIPTION	0.020302	5´ UTR
PbZn-miR-9	PADG_02543/ putative U6 snRNA phosphodiesterase	-22.9 kcal/mol	TRANSCRIPTION	0.045399	5´ UTR
PbZn-miR-9	PADG_02948/ nuclear RNA binding protein	-23.1 kcal/mol	TRANSCRIPTION	0.041559	5´ UTR
PbZn-miR-9	PADG_02996/ PAB1 binding protein	-25.4 kcal/mol	TRANSCRIPTION	0.015719	5´ UTR
PbZn-miR-9	PADG_03195/ KRR1 small subunit processome component	-23.0 kcal/mol	TRANSCRIPTION	0.043529	5´ UTR
PbZn-miR-9	PADG_03295/ HLH transcription factor	-26.4 kcal/mol	TRANSCRIPTION	0.010119	5´ UTR
PbZn-miR-9	PADG_03502/ C6 finger domain-containing protein	-25.1 kcal/mol	TRANSCRIPTION	0.017866	5´ UTR
PbZn-miR-9	PADG_03731/ fungal specific transcription factor	-23.7 kcal/mol	TRANSCRIPTION	0.032393	5´ UTR
PbZn-miR-9	PADG_03883/ Sin3 complex subunit	-23.5 kcal/mol	TRANSCRIPTION	0.035252	5´ UTR
PbZn-miR-9	PADG_03966/ HCNGP domain-containing protein	-24.4 kcal/mol	TRANSCRIPTION	0.024070	5´ UTR
PbZn-miR-9	PADG_04031/ tRNA (guanine-N(7)-)-methyltransferase non-catalytic subunit TRM82	-23.2 kcal/mol	TRANSCRIPTION	0.040012	5´ UTR
PbZn-miR-9	PADG_04039/ protein vts1	-24.5 kcal/mol	TRANSCRIPTION	0.022970	5´ UTR
PbZn-miR-9	PADG_04186/ SRF-type transcription factor RlmA	-23.5 kcal/mol	TRANSCRIPTION	0.035853	5´ UTR
PbZn-miR-9	PADG_04369/ splicing factor U2AF 23 kDa subunit	-23.5 kcal/mol	TRANSCRIPTION	0.035252	5´ UTR
PbZn-miR-9	PADG_04378/ DNA-directed RNA polymerase III subunit RPC6	-22.8 kcal/mol	TRANSCRIPTION	0.047347	5´ UTR
PbZn-miR-9	PADG_04436/ RNA-binding protein 8A	-22.7 kcal/mol	TRANSCRIPTION	0.049377	5´ UTR
PbZn-miR-9	PADG_04616/ smad nuclear-interacting protein 1	-22.7 kcal/mol	TRANSCRIPTION	0.049377	5´ UTR
PbZn-miR-9	PADG_04665/C2H2 finger domain-containing protein	-23.5 kcal/mol	TRANSCRIPTION	0.035252	5´ UTR
PbZn-miR-9	PADG_04672/ ATP-dependent RNA helicase SUB2	-23.0 kcal/mol	TRANSCRIPTION	0.042983	5´ UTR
PbZn-miR-9	PADG_05095/ Nucleolar protein 58	-23.9 kcal/mol	TRANSCRIPTION	0.029761	5´ UTR
PbZn-miR-9	PADG_05221/ Polyadenylation factor subunit 2	-22.7 kcal/mol	TRANSCRIPTION	0.049170	5´ UTR

PbZn-miR-9	PADG_05585/ Pre-mRNA-splicing factor syf1	-23.1 kcal/mol	TRANSCRIPTION	0.041734	5´ UTR
PbZn-miR-9	PADG_05597/ C6 finger domain transcription factor nscR	-23.0 kcal/mol	TRANSCRIPTION	0.043529	5´ UTR
PbZn-miR-9	PADG_05900/ Transcription elongation factor 1 homolog	-24.2 kcal/mol	TRANSCRIPTION	0.026205	5´ UTR
PbZn-miR-9	PADG_06433/ Transcription initiation factor TFIID subunit 8	-24.1 kcal/mol	TRANSCRIPTION	0.027341	5´ UTR
PbZn-miR-9	PADG_06697/ Poly(A) polymerase	-23.4 kcal/mol	TRANSCRIPTION	0.036774	5´ UTR
PbZn-miR-9	PADG_06824/C6 transcription factor	-25.1 kcal/mol	TRANSCRIPTION	0.017866	5´ UTR
PbZn-miR-9	PADG_06900/ RING finger domain-containing protein	-24.2 kcal/mol	TRANSCRIPTION	0.026205	5´ UTR
PbZn-miR-9	PADG_06912/ subunit of DNA polymerase II	-23.6 kcal/mol	TRANSCRIPTION	0.033226	5´ UTR
PbZn-miR-9	PADG_06916/ transcriptional regulation of mitochondrial recombination-domain-containing protein	-24.3 kcal/mol	TRANSCRIPTION	0.025008	5´ UTR
PbZn-miR-9	PADG_07126/ RING finger domain-containing protein	-23.6 kcal/mol	TRANSCRIPTION	0.033793	5´ UTR
PbZn-miR-9	PADG_07145/ CTD kinase subunit 3	-25.2 kcal/mol	TRANSCRIPTION	0.017119	5´ UTR
PbZn-miR-9	PADG_07267/ C6 finger domain transcription factor nscR	-24.1 kcal/mol	TRANSCRIPTION	0.027341	5´ UTR
PbZn-miR-9	PADG_07379/ transcription factor RfeG	-23.4 kcal/mol	TRANSCRIPTION	0.036774	5´ UTR
PbZn-miR-9	PADG_07483/ Histone acetyltransferase	-22.8 kcal/mol	TRANSCRIPTION	0.047149	5´ UTR
PbZn-miR-9	PADG_07509/ transcription initiation factor TFIID subunit 6	-22.7 kcal/mol	TRANSCRIPTION	0.049377	5´ UTR
PbZn-miR-9	PADG_07529/Queuine tRNA-ribosyltransferase accessory subunit 2	-23.3 kcal/mol	TRANSCRIPTION	0.038359	5´ UTR
PbZn-miR-9	PADG_07755/ Mediator of RNA polymerase II transcription subunit 11	-22.9 kcal/mol	TRANSCRIPTION	0.045399	5´ UTR
PbZn-miR-9	PADG_08069/ FACT complex subunit spt16	-27.2 kcal/mol	TRANSCRIPTION	0.007275	5´ UTR
PbZn-miR-9	PADG_08441/ FF domain-containing protein	-25.9 kcal/mol	TRANSCRIPTION	0.012695	5´ UTR
PbZn-miR-9	PADG_08499/ mRNA (guanine-N(7)-)-methyltransferase	-23.4 kcal/mol	TRANSCRIPTION	0.036774	5´ UTR
PbZn-miR-9	PADG_11068/ RNA interference and silencing protein	-23.9 kcal/mol	TRANSCRIPTION	0.029761	5´ UTR
PbZn-miR-9	PADG_11264/ C6 finger domain transcription factor nscR	-24.4 kcal/mol	TRANSCRIPTION	0.024070	5´ UTR
PbZn-miR-9	PADG_11344/ C2H2 finger domain-containing protein	-24.1 kcal/mol	TRANSCRIPTION	0.027341	5´ UTR
PbZn-miR-9	PADG_11352/ transcription factor RfeF	-24.6 kcal/mol	TRANSCRIPTION	0.022107	5´ UTR
PbZn-miR-9	PADG_11415/ RXT2-like protein	-23.2 kcal/mol	TRANSCRIPTION	0.040012	5´ UTR
PbZn-miR-9	PADG_11500/ C6 finger domain transcription factor nscR	-23.1 kcal/mol	TRANSCRIPTION	0.041734	5´ UTR
PbZn-miR-9	PADG_11586/ RNA-dependent RNA polymerase	-26.5 kcal/mol	TRANSCRIPTION	0.009820	5´ UTR
PbZn-miR-9	PADG_11710/ PWI domain-containing protein	-31.4 kcal/mol	TRANSCRIPTION	0.001197	5´ UTR
PbZn-miR-9	PADG_12197/ nucleolar protein NOP58	-24.1 kcal/mol	TRANSCRIPTION	0.027341	5´ UTR

PbZn-miR-9	PADG_12218/ 3' exoribonuclease	-25.0 kcal/mol	TRANSCRIPTION	0.018644	5´ UTR
PbZn-miR-9	PADG_12256/ fungal specific transcription factor domain-containing protein	-24.2 kcal/mol	TRANSCRIPTION	0.026205	5´ UTR
PbZn-miR-9	PADG_05322/ ZIP metal ion transporter (zrt3)	-23.5 kcal/mol	Transport	0.035252	5´ UTR
PbZn-miR-9	PADG_08196/ Vacuolar membrane zinc transporter (cot1)	-24.3 kcal/mol	Transport	0.025115	5´ UTR
PbZn-miR-9	PADG_04903/ Mitochondrial iron transporter	-24.2 kcal/mol	Transport	0.026205	3´ UTR
