# Supplementary material for: Global Molecular Response of Paracoccidioides brasiliensis to Zinc Deprivation: Analyses at Transcript, Protein and MicroRNA Levels
Source: J Fungi (Basel). 2023 Feb 21;9(3):281. doi: 10.3390/jof9030281 (PMC10056003; doi:10.3390/jof9030281)
Supplement: Supplementary file 1 [file jof-09-00281-s001.zip › Table S2.pdf]

Table S2. Potential mRNAs encoding proteins targets of PbZn-miR-4, repressed by zinc deprivation, related to cellular transport.

MicroRNA	Superconting	Targets	MFE	Functional classification	P-value	
PbZn-miR-4	Supercontig_2.14_28062	PADG_00180/Got1 family protein	-26.2 kcal/mol	Transport	0.014972	3'UTR
PbZn-miR-4	Supercontig_2.14_28062	PADG_01941/HIG1 domain-containing protein	-25.8 kcal/mol	Transport	0.017461	3'UTR
PbZn-miR-4	Supercontig_2.14_28062	PADG_02798/family integral membrane protein	-26.0 kcal/mol	Transport	0.016270	3'UTR
PbZn-miR-4	Supercontig_2.14_28062	PADG_03068/YeeE/YedE family integral membrane protein	-25.2 kcal/mol	Transport	0.023056	3'UTR
PbZn-miR-4	Supercontig_2.14_28062	PADG_03862/RING-type domain-containing protein	-24.6 kcal/mol	Transport	0.029065	3'UTR
PbZn-miR-4	Supercontig_2.14_28062	PADG_04027/endoplasmic reticulum-Golgi intermediate compartment protein	-23.7 kcal/mol	Transport	0.042097	3'UTR
PbZn-miR-4	Supercontig_2.14_28062	PADG_04037/MFS multidrug transporter	-24.0 kcal/mol	Transport	0.037217	3'UTR
PbZn-miR-4	Supercontig_2.14_28062	PADG_04160/MRS7 family protein	-25.3 kcal/mol	Transport	0.021756	3'UTR
PbZn-miR-4	Supercontig_2.14_28062	PADG_04327/MFS multidrug transporter	-25.4 kcal/mol	Transport	0.020873	3'UTR
PbZn-miR-4	Supercontig_2.14_28062	PADG_04690/vesicle-associated membrane protein 4	-24.1 kcal/mol	Transport	0.035425	3'UTR
PbZn-miR-4	Supercontig_2.14_28062	PADG_04861/rhomboid family protein	-25.6 kcal/mol	Transport	0.019211	3'UTR
PbZn-miR-4	Supercontig_2.14_28062	PADG_04883/ Protein transporter SEC24	-23.6 kcal/mol	Transport	0.043859	3'UTR
PbZn-miR-4	Supercontig_2.14_28062	PADG_06348/MFS transporter	-23.9 kcal/mol	Transport	0.038779	3'UTR
PbZn-miR-4	Supercontig_2.14_28062	PADG_07230/TPR_REGION domain-containing protein	-24.4 kcal/mol	Transport	0.031565	3'UTR
PbZn-miR-4	Supercontig_2.14_28062	PADG_07960/tetraspanin	-28.0 kcal/mol	Transport	0.007070	3'UTR
PbZn-miR-4	Supercontig_2.14_28062	PADG_08567/zinc-regulated transporter 1 (ZRT1)	-23.6 kcal/mol	Transport	0.043859	3'UTR
PbZn-miR-4	Supercontig_2.14_28062	PADG_08687/Major facilitator superfamily domain, general substrate transporter	-23.4 kcal/mol	Transport	0.047600	3'UTR
PbZn-miR-4	Supercontig_2.14_28062	PADG_11487/calcium transporter	-24.9 kcal/mol	Transport	0.025676	3'UTR
PbZn-miR-4	Supercontig_2.14_28062	PADG_11514/Sodium/metabolite cotransporter like	-25.4 kcal/mol	Transport	0.020873	3'UTR
PbZn-miR-4	Supercontig_2.14_28062	PADG_11722/sucrose transport protein	-27.3 kcal/mol	Transport	0.009469	3'UTR
PbZn-miR-4	Supercontig_2.14_28062	PADG_11728/AP-1 complex subunit beta-1	-25.1 kcal/mol	Transport	0.023636	3'UTR
PbZn-miR-4	Supercontig_2.14_28062	PADG_00470/ Ferric oxidoreductase domain-containing protein	-23.4 kcal/mol	Transport	0.047600	5'UTR
PbZn-miR-4	Supercontig_2.14_28062	PADG_01298/ EF hand domain-containing protein	-23.3 kcal/mol	Transport	0.049384	5'UTR
PbZn-miR-4	Supercontig_2.14_28062	PADG_01471/ S-adenosylmethionine-dependent methyltransferase	-25.4 kcal/mol	Transport	0.021047	5'UTR
PbZn-miR-4	Supercontig_2.14_28062	PADG_01528/ endoplasmic reticulum vesicle protein 25	-24.5 kcal/mol	Transport	0.030290	5'UTR

PbZn-miR-4	Supercontig_2.14_28062	PADG_01662/ plasma membrane fusion protein PRM1	-24.0 kcal/mol	Transport	0.037217	5'UTR
PbZn-miR-4	Supercontig_2.14_28062	PADG_02020/ syntaxin 16	-23.3 kcal/mol	Transport	0.049586	5'UTR
PbZn-miR-4	Supercontig_2.14_28062	PADG_02058/ von Willebrand and RING finger domain-containing protein	-23.9 kcal/mol	Transport	0.039421	5'UTR
PbZn-miR-4	Supercontig_2.14_28062	PADG_02884/ calcium channel subunit Cch1	-23.4 kcal/mol	Transport	0.047600	5'UTR
PbZn-miR-4	Supercontig_2.14_28062	PADG_04021/ MFS monocarboxylate transporter	-23.4 kcal/mol	Transport	0.047406	5'UTR
PbZn-miR-4	Supercontig_2.14_28062	PADG_04941/ metal homeostasis protein bsd2	-27.2 kcal/mol	Transport	0.009872	5'UTR
PbZn-miR-4	Supercontig_2.14_28062	PADG_06466/ transmembrane protein UsgS	-30.8 kcal/mol	Transport	0.002192	5'UTR
PbZn-miR-4	Supercontig_2.14_28062	PADG_06814/ protein SSH4	-25.2 kcal/mol	Transport	0.022677	5'UTR
PbZn-miR-4	Supercontig_2.14_28062	PADG_06817/ translocation protein sec63	-24.8 kcal/mol	Transport	0.026760	5'UTR
PbZn-miR-4	Supercontig_2.14_28062	PADG_07440/ dicarboxylic amino acid permease	-23.6 kcal/mol	Transport	0.043859	5'UTR
PbZn-miR-4	Supercontig_2.14_28062	PADG_07610/ MFS transporter	-23.3 kcal/mol	Transport	0.049586	5'UTR
PbZn-miR-4	Supercontig_2.14_28062	PADG_08632/ ESCRT-II complex subunit VPS36	-23.6 kcal/mol	Transport	0.043859	5'UTR
PbZn-miR-4	Supercontig_2.14_28062	PADG_11819/ GTPase-activating protein GYP7	-24.9 kcal/mol	Transport	0.025676	5'UTR
PbZn-miR-4	Supercontig_2.14_28062	PADG_12160/ auxin Efflux Carrier superfamily	-23.4 kcal/mol	Transport	0.047600	5'UTR
