# Supplementary material for: Global Molecular Response of Paracoccidioides brasiliensis to Zinc Deprivation: Analyses at Transcript, Protein and MicroRNA Levels
Source: J Fungi (Basel). 2023 Feb 21;9(3):281. doi: 10.3390/jof9030281 (PMC10056003; doi:10.3390/jof9030281)
Supplement: Supplementary file 1 [file jof-09-00281-s001.zip › Table S3.pdf]

Table S3. Proteins decreased at 24 hours of zinc deprivation and their functional classification.

Accession Numbers	Protein designation	Score	Fold change	Metals
METABOLISM				
amino acid metabolism				
PADG_01928	S-adenosylmethionine synthase	2873.68	0.477113911	K, Mg, Co, Ca, Na
PADG_06319	Glutamate decarboxylase	533.91	*	Na
PADG_01564	Methylmalonate-semialdehyde dehydrogenase (Acylating)	655.53	*	K, Mg, Rb
PADG_05492	serine 3-dehydrogenase	1010.48	*	Na
PADG_06756	Histidine biosynthesis trifunctional protein	663.29	*	Zn, Cd
PADG_01718	Saccharopine dehydrogenase [NADP+, L-glutamate-forming]	744.64	*	
PADG_02347	carboxypeptidase D	1159.76	*	Ni
PADG_00211	glycine dehydrogenase	1352.81	*	
PADG_02777	Threonine synthase	1177.76	*	
PADG_03149	Aminopeptidase	682.03	*	Zn, K, Mg, Ca, Na
nitrogen, sulfur and selenium metabolism				
PADG_07010	Urease accessory protein UreG	592.24	*	Mg. Zn, Ni
PADG_11833	amidohydrolase	1071.23	*	Hg, Na, Mg, As
nucleotide/nucleoside/nucleobase metabolism				
PADG_04828	Adenylosuccinate lyase	929.76	0.26447725	Ca, K, Mg
PADG_04478	Polynucleotide kinase 3'-phosphatase	635.23	*	Mn, Mg
C-compound and carbohydrate metabolism				
PADG_02719	dTDP-4-dehydrorhamnose reductase	634.61	*	Mg, Ca
Threonine	UDP-galactopyranose mutase	700.81	*	
lipid, fatty acid and isoprenoid metabolism				
PADG_12025	glutaryl-CoA dehydrogenase	586.5	*	Mg, Na, Ca
PADG_03449	Isopentenyl-diphosphate delta-isomerase	641.37	*	Mn, Mg, Na
PADG_01228	3-hydroxybutyryl-CoA dehydrogenase	504.6	*	

metabolism of vitamins, cofactors, and prosthetic groups				
PADG_05462	pyridoxamine 5'-phosphate oxidase	618.57	*	
secondary metabolismo				
PADG_05310	Leukotriene A(4) hydrolase	651.48	*	Yb, Zn
PADG_04184	2OG-Fe(II) oxygenase	534.39	*	Fe, Mn, Ca, Ni, Zn
ENERGY				
glycolysis and gluconeogenesis				
PADG_05109	2,3-bisphosphoglycerate-independent phosphoglycerate mutase	664.84	0.193980045	Mn, Co, Na, Zn, Mg
PADG_03631	phosphoglycerate mutase family protein	796.38	*	
PADG_02063	Pyruvate dehydrogenase (Acetyl-transferring) E1 component, alpha subunit	544.78	*	K, Mg, Mn, Na
PADG_04687	Glucose 1-dehydrogenase	1840.84	*	Mg, Zn
tricarboxylic-acid pathway (citrate cycle, Krebs cycle, TCA cycle)				
PADG_08119	Fumarate hydratase, mitochondrial	535.55	*	Na, Co, Ca
electron transport and membrane-associated energy conservation				
PADG_05343	NADH-ubiquinone oxidoreductase 21.3 kDa subunit	549.32	*	
PADG_06196	12-oxophytodienoate reductase	2627.36	0.65050909	Mg, Na
PADG_00408	cytochrome C	1044.12	0.244143291	
PADG_00893	F0F1 ATP synthase subunit gamma	626.11	*	
PADG_04559	Cytochrome b5 heme-binding domain-containing protein	5040.49	*	Fe
PADG_08460	NADPH dehydrogenase	1123.64	*	Fe, Ca
PADG_00052	Succinate dehydrogenase [ubiquinone] flavoprotein subunit, mitochondrial	785.93	*	K, Ca, Na
Fermentation				
PADG_11405	Alcohol dehydrogenase 1	4049.86	0.045501958	Zn
PADG_04701	alcohol dehydrogenase	753.53	*	As, Zn, Mg, Mn, K
oxidation of fatty acids				
PADG_04687	Short-chain dehydrogenase	1840.84	*	Mg, Zn
CELL CYCLE AND DNA PROCESSING				

PADG_01688	DlpA domain-containing protein	908.41	*	Mg
PADG_00466	mitochondrial genome maintenance protein mgm101	1218.52	*	
PADG_05798	ssDNA binding protein, putative	8976.85	0.663650253	Ca
PADG_05157	period circadian protein	659.73	*	
TRANSCRIPTION				
PADG_05587	U2 small nuclear ribonucleoprotein B	852.23	*	
PROTEIN SYNTHESIS				Ca, Mg, Na
ribosome biogenesis				
PADG_12027	ribosomal RNA small subunit methyltransferase NEP1	609.51	*	
PADG_01387	60S ribosomal protein L7	846.83	*	
PADG_02249	60S ribosomal protein L2	2277.57	*	
PADG_12365	40S ribosomal protein S8	745.03	*	
PADG_00046	mitochondrial ribosomal protein L37	968.54	*	
PADG_03767	Ribosomal RNA small subunit methyltransferase	573.08	*	
Translation				
PADG_08424	CD2 antigen cytoplasmic tail-binding protein 2	964.19	*	
PADG_11711	ATP-dependent RNA helicase eIF4A	924.69	*	
PADG_07888	Eukaryotic translation initiation factor 5A	920.62	*	Zn, Mg, Al, Be, W
PADG_05642	Signal recognition particle, SRP9	747.2	*	
PROTEIN FATE (folding, modification, destination)				
protein folding and stabilization				
PADG_03211	Protein ROT1	613.03	*	
protein modification				Mn, Zn, Ca, Mg, As, Na, Ca, Cd
C1GEY4	Probable Xaa-Pro aminopeptidase	841.88	*	
PADG_03913	Serine/threonine-protein kinase	2303.12	*	Zn
PADG_01086	chaperone protein dnaJ 3	800.24	*	
protein/peptide degradation				Ni, Pb, Na, Mg, Mn, Cd, Sm, Zn
PADG_01852	Hsc70 cochaperone (SGT), putative	610.66	0.527292432	K

PADG_06290	Proteasome endopeptidase complex	1002.35	*	Zn, Mg, Co, As
PADG_04167	aspartyl aminopeptidase	1309.5	0.663650253	Mg
PADG_03967	proteasome component C5	591.98	*	
PROTEIN WITH BINDING FUNCTION OR COFACTOR REQUIREMENT (structural or catalytic)				
nucleic acid binding				
PADG_07670	SAP domain protein	831.4	*	
PADG_11345	mariner-Tc1 transposon family protein	687.24	*	
CELLULAR TRANSPORT, TRANSPORT FACILITIES AND TRANSPORT ROUTES				
PADG_02022	Clathrin light chain	676.86	*	
PADG_02134	Coatomer subunit epsilon	617.02	*	
REGULATION OF METABOLISM AND PROTEIN FUNCTION				Ca, Mg, Ni, Na
PADG_02017	Calmodulin	1582.71	*	
CELLULAR COMMUNICATION/SIGNAL TRANSDUCTION MECHANISM				
PADG_03120	RIC8	524.98	*	
PADG_01285	gamma-aminobutyric acid A receptor	636.29	*	
PADG_07506	Protein phosphatase inhibitor 2	2068.58	*	
CELL RESCUE, DEFENSE AND VIRULENCE				
stress response				
PADG_04907	Hsp9/Hsp12 family heat shock protein	579.98	*	
Detoxification				
PADG_07627	4-carboxymuconolactone decarboxylase family protein	923.04	*	
PADG_01551	Thioredoxin reductase	938.13	*	Mg, Au, Na, Cd, Ca, K, As
PADG_03276	S-(hydroxymethyl)glutathione dehydrogenase	675.68	*	Zn, K, Cd, Cu
PADG_03403	aldehyde dehydrogenase	737.52	*	Mn, Mg, Na
CELL FATE				
PADG_06568	TCTP family protein	2990.31	0.576949804	
PADG_04966	phosducin family protein	725.9	*	Na

BIOGENESIS OF CELLULAR COMPONENTS			
cytoskeleton/structural proteins			
PADG_05893	myosin-2	542.56	*
PADG_05239	Tubulin-specific chaperone A	740.19	*
UNCLASSIFIED PROTEINS			
PADG_11913	Uncharacterized protein	1706.2	*
PADG_08409	Uncharacterized protein	1570.85	*
PADG_12242	Uncharacterized protein	751.42	*
PADG_06179	Uncharacterized protein	548.48	*

* Proteins found only under the control condition.
