# Supplementary material for: Global Molecular Response of Paracoccidioides brasiliensis to Zinc Deprivation: Analyses at Transcript, Protein and MicroRNA Levels
Source: J Fungi (Basel). 2023 Feb 21;9(3):281. doi: 10.3390/jof9030281 (PMC10056003; doi:10.3390/jof9030281)
Supplement: Supplementary file 1 [file jof-09-00281-s001.zip › Table S4.pdf]

Table S4. Proteins induced after 24 hours of zinc deprivation and their functional classification.

Accession P. brasiliensis	Protein	Score	Fold change	Metals
METABOLISM				
amino acid metabolismo				
PADG_08034	dienelactone hydrolase family protein	683.21	*	
PADG_01404	Aspartate aminotransferase	1169.64	*	Mg, As
PADG_03686	Aspartate aminotransferase	642.36	*	Mg, As
PADG_08304	Acetolactate synthase, small subunit	558.56	*	Mg, Ca
PADG_03627	2-oxoisovalerate dehydrogenase subunit beta	1233.24	*	K, Mg
PADG_02591	amidase	1183.16	*	Cd
PADG_05277	Serine hydroxymethyltransferase	924.36	*	
PADG_01886	Adenosylhomocysteinase	2822.8	1.537257535	Na,
PADG_04718	2-methylcitrate dehydratase	12853.83	1.537257535	
PADG_05301	Cystathionine beta-synthase	1035.09	*	Fe, Zn, Na
PADG_02625	4-coumarate-CoA ligase	696.68	*	Mg, Ca
nucleotide/nucleoside/nucleobase metabolism				
PADG_07585	Inosine-5'-monophosphate dehydrogenase	610.78	*	K, Mn, Mg
phosphate metabolismo				
PADG_05822	Pyridoxine biosynthesis protein pyroA	1499.88	2.075080647	Mg, As
lipid, fatty acid and isoprenoid metabolism				
PADG_05783	farnesyl pyrophosphate synthetase	643.26	5.989452238	Mg, Zn
PADG_06876	3-hydroxyisobutyryl-CoA hydrolase, mitochondrial	2433.19	2.054433269	Mg, Ca, Zn
metabolism of vitamins, cofactors, and prosthetic groups				
PADG_00443	Dihydropteroate synthase	971.68	1.521961536	Na
PADG_05474	thiamin pyrophosphokinase-related protein	658.69	*	
ENERGY				
glycolysis and gluconeogenesis				
PADG_00451	Glucose-6-phosphate isomerase	549.25	2.386910865	Ca, Na

PADG_04165	pyruvate dehydrogenase complex component Pdx1	901.91	3.095656485	
PADG_03813	Phosphotransferase	1815.11	*	Ca, K, Mg, Na
PADG_03118	Glucose-6-phosphate 1-epimerase	2313.56	1.786038401	Na, Ba
PADG_01278	Pyruvate kinase	2588.75	1.768267039	K, Mn, Mg,
PADG_08503	Phosphoenolpyruvate carboxykinase [ATP]	924.76	1.521961536	Mg, Mn, Ca, Al, Na
PADG_01896	Phosphoglycerate kinase	700.89	1.896480852	Mg, Na, Ca, Al,K,Be
PADG_00714	Pyruvate decarboxylase	1271.04	3.896193358	Mg
pentose-phosphate pathway				
PADG_03651	6-phosphogluconate dehydrogenase, decarboxylating	794.91	*	As
PADG_04604	Transketolase	769.52	1.973877746	Ca, Mg, Na
PADG_07771	6-phosphogluconolactonase	1307.77	1.858928051	Hg ,Zn, K, Na, Cu
tricarboxylic-acid pathway (citrate cycle, Krebs cycle, TCA cycle)				
PADG_05904	dihydrolipoamide succinyltransferase	706.2	*	Mg, Ca
PADG_02805	Isocitrate dehydrogenase [NAD] subunit, mitochondrial	778.35	*	Ca, Zn, Mg, Na, Mn
PADG_02260	Succinate--CoA ligase [ADP-forming] subunit alpha, mitochondrial	700.26	1.858928051	Mg, Na
electron transport and membrane-associated energy conservation				
PADG_04501	Cytochrome b-c1 complex subunit 7	1785.44	1.993715528	
PADG_03559	cytochrome b5	1158.52	2.316366916	Fe, Mg, Ca, Na, Cu, Zn
PADG_07042	ATP synthase F1, delta subunit	826.59	*	
PADG_02578	ATP synthase subunit 4, mitochondrial	637.32	*	
PADG_11513	NADH-ubiquinone oxidoreductase	729	*	
PADG_08394	cytochrome b-c1 complex subunit 2	674.27	*	Fe, Mg, Ca, Na, Cu
PADG_05290	cytochrome b5	631.58	*	Fe, Mg, Ca, Na, Cu
PADG_00171	cytochrome b2	922.14	1.858928051	Fe, Mg,
PADG_07813	ATP synthase subunit gamma	628.12	1.665291179	
PADG_11657	ATP-binding protein	4778.75	*	

PADG_08152	cytochrome oxidase c	1134.4	*	
PADG_06766	Mitochondrial-processing peptidase subunit beta	577.46	1.750672504	Zn
Respiration				
PADG_04099	Phosphoribosylaminoimidazolecarboxamide formyltransferase/IMP cyclohydrolase	2320.17	2.013752683	K, Mg
metabolism of energy reserves (e.g. glycogen, trehalose)				
PADG_02145	Alpha-1,4 glucan phosphorylase	719.83	*	V
oxidation of fatty acids				
PADG_01687	3-ketoacyl-CoA thiolase	2957.83	2.247907992	Na, K
PADG_08651	Peroxisomal hydratase-dehydrogenase-epimerase	641.95	*	Na
CELL CYCLE AND DNA PROCESSING				
PADG_04869	HIT domain protein	3516.93	2.386910865	W, Zn, Mg, Na, Cu
PADG_01032	DNA-binding protein, 42 kDa	1010.9	*	Co, Zn, Mn
PADG_04056	14-3-3 family protein epsilon	3641.54	1.934792385	Mg, Ni, As
PADG_06942	Kinetochore protein SPC24	671.36	*	
PADG_06986	calcineurin binding protein	685.08	*	
PADG_02300	phosphatase 2C Ptc3	708.74	*	Mn, Zn, Ca, Mg, Pr
PADG_05683	Cell division control protein 48	1040.03	3.158192834	Mg
TRANSCRIPTION				
PADG_02473	pirin	797.42	3.034358438	Cd, Fe, Co, Mn
PADG_03696	nuclear polyadenylated RNA-binding protein Nab2	1151.31	*	Zn
PADG_06898	U1 small nuclear ribonucleoprotein 70 kDa	1014.36	*	Mg
PADG_08036	nmrA-like family	673.95	*	Cu, Mg
PADG_01442	protein STB3	561.21	1.632316236	
PROTEIN SYNTHESIS				
PADG_00824	telomere and ribosome associated protein Stm1	772.23	*	
ribosome biogenesis				
PADG_02445	40S ribosomal protein S15	2011.43	1.993715528	
PADG_01407	40S ribosomal protein	1188.06	2.033991215	

C1G1P4	40S ribosomal protein S0	1323.72	2.117000017	
PADG_02056	Ribosomal protein L7/L12	1663.69	9.393331377	
PADG_11413	60S ribosomal protein L9	3008.07	*	
PADG_08605	40S ribosomal protein S28	1235.95	2.773194711	
PADG_08238	40S ribosomal protein S26	626.97	*	Zn
PADG_06313	40S ribosomal protein S18	819.2	*	
PADG_04106	60S ribosomal protein L11	789.58	*	
PADG_05883	60S ribosomal protein L25	1150.04	1.915540783	
PADG_12253	60S ribosomal protein L3	731.56	1.858928051	
PADG_08715	60S ribosomal protein L28	4593.55	1.768267039	
PADG_07870	30S ribosomal protein S7	746.14	1.632316236	
PADG_02446	60S acidic ribosomal protein P2	5483.59	1.599994191	
PADG_08602	40S ribosomal protein S2	838.59	1.521961536	
PADG_04475	Ribosomal protein S36, mitochondrial	588.43	*	
Translation				
PADG_01949	Elongation factor Tu	3095.05	1.973877746	Mg,
PADG_08125	Elongation factor 2	2954.14	2.203396474	Mg
PADG_02759	ribosome recycling factor domain-containing protein	2606.2	2.363160728	Hg
C1GJ45	Eukaryotic translation initiation factor 3 subunit A	1263.28	*	Al, Mg, Cs
PADG_07977	Eukaryotic translation initiation factor 1A, Y-chromosomal	782.54	*	
PADG_06265	elongation factor 1 gamma domain-containing protein	9834.64	1.87761057	Cd, K, Na, Co
PADG_00692	Elongation factor 1-alpha	2644.04	1.750672504	Mg
PADG_04210	translation initiation factor 2 subunit beta	1405.18	1.716006899	Zn
PADG_07884	Polyadenylate-binding protein	848.27	2.410899695	Cd, Zn
PADG_06160	eukaryotic translation initiation factor 2 subunit alpha	713.96	*	Zn
PADG_01160	Eukaryotic translation initiation factor 3 subunit M	731.71	*	
aminoacyl-tRNA-synthetases				
PADG_02484	Valine-tRNA ligase	713.25	*	Mg, Zn, Hg
PADG_04962	Aspartate-tRNA(Asn) ligase	607.02	1.822118844	Mg, Mn, Na

PADG_01558	histidyl-tRNA synthetase	1252.2	1.648721271	W, Na
PROTEIN FATE (folding, modification, destination)				
PADG_12323	Peptidyl-prolyl cis-trans isomerase	913.64	*	Au
protein modification				
PADG_02637	ubiquitin-conjugating enzyme	631.98	*	Na, Ca, K, Co, Zn
protein/peptide degradation				
PADG_04076	Proteasome subunit beta type	758.58	*	
PADG_11128	26S protease regulatory subunit 6B	653.99	*	Mg
PADG_04088	zinc metalloprotease	868.12	*	Mg, Zn, Co, As, Ca
PADG_12469	26S proteasome non-ATPase regulatory subunit 6	685.76	*	
PROTEIN WITH BINDING FUNCTION OR COFACTOR REQUIREMENT (structural or catalytic)				
metal binding				
PADG_01717	cupin domain-containing protein	942.91	*	
C1FYL2	Fe-S cluster assembly protein DRE2	810.7	*	Mg
CELLULAR TRANSPORT, TRANSPORT FACILITIES AND TRANSPORT ROUTES				
PADG_07508	phosphatidylinositol transfer protein SFH5	1074.3	2.386910865	
PADG_02199	transporter of Nicotinic Acid	1743.85	*	
PADG_01243	Rab GDP dissociation inhibito	1188.11	1.616074385	Mn, Zn, Ca, Pr, Mg
PADG_06165	glycolipid transferprotein HET-C2	639.09	*	Ni, Na
PADG_02619	ER protein translocation	732.21	*	
PADG_07714	NTF2 and RRM domain protein	1134.38	2.054433269	K
CELLULAR COMMUNICATION/SIGNAL TRANSDUCTION MECHANISM				
PADG_05608	GTP-binding protein ypt7	1135.71	*	Mg, Sr, K, Al, Be, Y
PADG_00282	GTP-binding protein ypt2	678.73	*	Co, Mg, Ba, Al, Be, Zn
PADG_07281	Guanine nucleotide-binding protein subunit alpha	722.24	*	Al, Mg, Cs

CELL RESCUE, DEFENSE AND VIRULENCE				
stress response				
PADG_05183	Glutaredoxin	1817.33	*	Fe, Na
PADG_07477	ankyrin repeat protein (Yar1)	747.51	*	
PADG_05032	Hsp90 binding co-chaperone (Sba1)	837.22	1.840431425	
PADG_08369	Hsp60-like protein	17540.67	1.822118844	Tl, Mg, Be, K
PADG_01711	Hsp90 co-chaperone AHA1	2639.66	1.632316236	
PADG_07715	Hsp90-like protein	4690.84	1.632316236	Cd, Mg
PADG_07953	Peptidyl-prolyl cis-trans isomerase	1115.09	1.568312167	Ca, Zn, Ni, Na, Cd
PADG_02761	Hsp75-like protein	2552.27	1.50681778	Mg, Na, Ca, K, Zn, Mn, W
PADG_05081	Aldehyde dehydrogenase	1213.69	4.903749092	Na, Mg
PADG_02030	Hsp90 co-chaperone Cdc37	3131.07	2.159766213	
CELL FATE				
PADG_05491	GMF family protein	681.93	*	
BIOGENESIS OF CELLULAR COMPONENTS				
cytoskeleton/structural proteins				
PADG_00945	Actin-related protein 2/3 complex subunit 5	760.69	*	
PADG_11857	mitotic checkpoint protein BUB3	828.18	*	Ca, Mg
PADG_12087	kinesin heavy chain	1366.45	*	
PADG_12076	Actin	1136.22	*	Mg, Ca
PADG_00128	Tubulin alpha chain	717.7	1.840431425	Mg
PADG_00422	actin cytoskeleton protein (VIP1)	4990.33	1.750672504	
PADG_08368	cofilin	1170.43	1.616074385	
cell wall				
PADG_06336	cell lysis protein cwl1	761.78	1.716006899	
PADG_06759	putative chitin synthase	697.53	*	
Golgi				

PADG_00463	DUF833 domain-containing protein	708.37	*	
Mitochondrion				
PADG_04242	Sulfhydryl oxidase	746.32	*	Z, Na, Cd
UNCLASSIFIED PROTEINS				
PADG_00674	Uncharacterized protein	956.35	4.220696059	
PADG_02338	Uncharacterized protein	2030.95	2.075080647	
PADG_07617	Uncharacterized protein	871.57	*	
PADG_11975	Uncharacterized protein	711.97	*	
PADG_05780	Uncharacterized protein	775.21	*	
PADG_12023	Uncharacterized protein	1307.21	*	
PADG_00939	Uncharacterized protein	1155.7	*	

* Proteins found only under the under-zinc deprivation condition.
