# Supplementary material for: Global Molecular Response of Paracoccidioides brasiliensis to Zinc Deprivation: Analyses at Transcript, Protein and MicroRNA Levels
Source: J Fungi (Basel). 2023 Feb 21;9(3):281. doi: 10.3390/jof9030281 (PMC10056003; doi:10.3390/jof9030281)
Supplement: Supplementary file 1 [file jof-09-00281-s001.zip › Table S5.pdf]

Table S5. Number of sequencing reads mapped in each replicate of the conditions used in the study.

Sample	Number of Readings
C1	26556055
C2	24016136
C3	21406924
D1	23619046
D2	23116878
D3	24295698
