# Supplementary material for: Global Molecular Response of Paracoccidioides brasiliensis to Zinc Deprivation: Analyses at Transcript, Protein and MicroRNA Levels
Source: J Fungi (Basel). 2023 Feb 21;9(3):281. doi: 10.3390/jof9030281 (PMC10056003; doi:10.3390/jof9030281)
Supplement: Supplementary file 1 [file jof-09-00281-s001.zip › Table S6.pdf]

Table S6. Transcripts induced during zinc deprivation.

GenBank Accession *P. brasiliensis*	Protein	Metals	Fold change
METABOLISM			
amino acid metabolism			
PADG_01834	GGACT domain-containing protein		0.5980568
PADG_03513	Methyltransf_11 domain-containing protein	Na	0.5931847
PADG_05198	Pyridoxal 5'-phosphate synthase, glutaminase subunit Pdx2		0.6175450
ENERGY			
glycolysis and gluconeogenesis			
PADG_03071	PEP phosphonomutase		0.6640193
electron transport and membrane-associated energy conservation			
PADG_08666	ATP synthase subunit K		1
TRANSCRIPTION			
PADG_07162	U6 snRNA-associated Sm-like protein LSm4		0.6442036
PADG_12476	Nucleoside triphosphate pyrophosphohydrolase		0.6213017
PADG_07993	pre-mRNA splicing factor	Ni, Mg, K	0.5997856
PROTEIN SYNTHESIS			
ribosome biogenesis			
PADG_04588	60S ribosomal protein L22		0.6558204
Translation			
PADG_00806	BTB domain-containing protein		0.6141178
PADG_12047	tRNA-Phe		0.7027423
aminoacyl-tRNA-synthetases			
PADG_05841	peptidyl-tRNA hydrolase		0.5949724
PROTEIN FATE (folding, modification, destination)			

PADG_00806	Kelch-like protein 38		0.6141178
PADG_12469	26S proteasome non-ATPase regulatory subunit 6		0.6218737
PROTEIN WITH BINDING FUNCTION OR COFACTOR REQUIREMENT (structural or catalytic)			
nucleic acid binding			
PADG_12474	C2H2 finger domain-containing protein		0.6910646
CELLULAR TRANSPORT, TRANSPORT FACILITIES AND TRANSPORT ROUTES			
PADG_06417	zinc-regulated transporter 2 (ZRT2)	Zn	0.8711713
PADG_06430	pyridoxal-5'-phosphate-dependent enzyme	Mg, Ca, Na, K, Mn	0.6199204
CELL RESCUE, DEFENSE AND VIRULENCE			
PADG_12219	TRI7		0.6045995
UNCLASSIFIED PROTEINS			
PADG_01117	Uncharacterized protein		0.6219716
PADG_04857	Uncharacterized protein		0.7014952
PADG_08487	Uncharacterized protein		1
PADG_11055	Uncharacterized protein		0.7078537
PADG_12475	Uncharacterized protein		0.6403045
