# Supplementary material for: Global Molecular Response of Paracoccidioides brasiliensis to Zinc Deprivation: Analyses at Transcript, Protein and MicroRNA Levels
Source: J Fungi (Basel). 2023 Feb 21;9(3):281. doi: 10.3390/jof9030281 (PMC10056003; doi:10.3390/jof9030281)
Supplement: Supplementary file 1 [file jof-09-00281-s001.zip › Table S7.pdf]

Table S7. Transcripts repressed during zinc deprivation.

GenBank Accession *P. brasiliensis* (Pb18)	Protein	Metals	Fold change
METABOLISM			
amino acid metabolism			
PADG_03859	Putative sorbitol (alcohol)-dehydrogenase	Zn, Cd, Na, Ca, Mn	-0.6763895
PADG_04516	Glutamate dehydrogenase	K	-0.7372061
nucleotide/nucleoside/nucleobase metabolism			
PADG_01017	CAF1 family ribonuclease		-0.6665615
PADG_02839	O-methyltransferase	Ca, Zn, Mg, K, Ni, Na, Mn	-0.6043833
ENERGY			
electron transport and membrane-associated energy conservation			
PADG_03258	lipid A export ATP-binding/permease protein msbA	Na, V, Hg, Mg	-0.7489959
PADG_04572	SCY1 protein kinase		-0.6092240
Fermentation			
PADG_01174	alcohol dehydrogenase	Zn	-0.8771634
CELL CYCLE AND DNA PROCESSING			
PADG_07768	UV excision repair protein		-0.5859608
PADG_11749	DNA-directed RNA polymerase II 138 Kda polypeptide		-0.6142618
PADG_11750	CCCH zinc finger DNA binding protein		-0.6840303
TRANSCRIPTION			
PADG_00411	C6 transcription factor (Ctf1B)		-0.8671628
PADG_02012	3-deoxy-7-phosphoheptulonate synthase		-0.6316335
PADG_04314	hemagglutinin protein		-0.7204081
PADG_04600	AP-1-like transcription factor		-0.6805913
PADG_05470	C6 transcription factor		-0.6195290
PROTEIN FATE (folding, modification, destination)			

protein folding and stabilization			
PADG_00517	DnaJ domain-containing protein		-0.6413279
CELLULAR TRANSPORT, TRANSPORT FACILITIES AND TRANSPORT ROUTES			
PADG_02707	Mg2 transporter protein CorA family protein		-0.5924410
PADG_04197	ABC transporter CDR4	Mg, Na , Ni, Pt	-1
CELLULAR COMMUNICATION/SIGNAL TRANSDUCTION MECHANISM			
PADG_06708	F-box domain containing protein		-0.7947586
PADG_07981	ankyrin repeat domain protein, putative	Mg, Na	-0.5957269
CELL RESCUE, DEFENSE AND VIRULENCE			
PADG_12387	fluconazole resistance protein		-1
BIOGENESIS OF CELLULAR COMPONENTS			
PADG_01807	integral membrane protein		-0.7176654
PADG_03130	Myosin-10		-0.6687912
PADG_04499	cell wall protein ECM33 precursor		-1
UNCLASSIFIED PROTEINS			
PADG_02365	Uncharacterized protein		-0.6620554
PADG_03348	Uncharacterized protein		-0.8722472
PADG_05949	Uncharacterized protein		-0.7440966
PADG_11100	Uncharacterized protein		-1
PADG_11724	Uncharacterized protein		-0.7259224
PADG_12424	Uncharacterized protein		-0.8216202
