# Supplementary figures and images for: Global Molecular Response of Paracoccidioides brasiliensis to Zinc Deprivation: Analyses at Transcript, Protein and MicroRNA Levels
Source: J Fungi (Basel). 2023 Feb 21;9(3):281. doi: 10.3390/jof9030281 (PMC10056003; doi:10.3390/jof9030281)

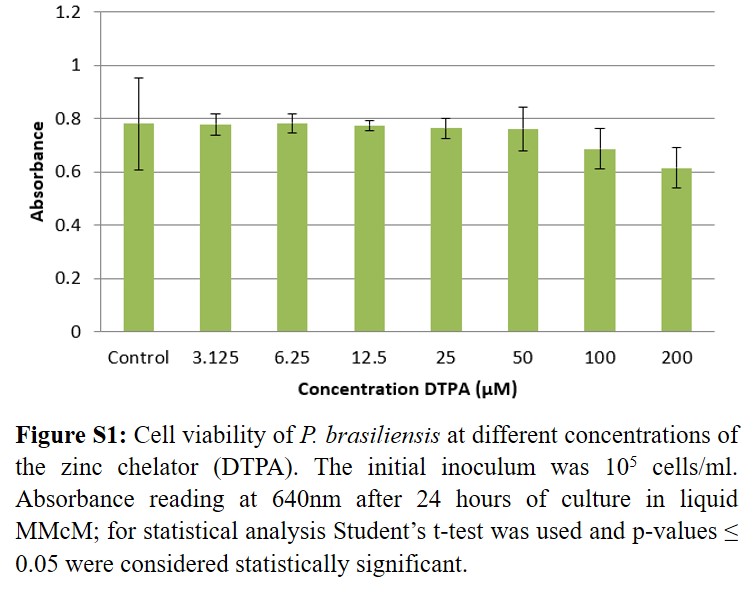

Supplement: Supplementary file 1 [file jof-09-00281-s001.zip › Figura S1.jpg]

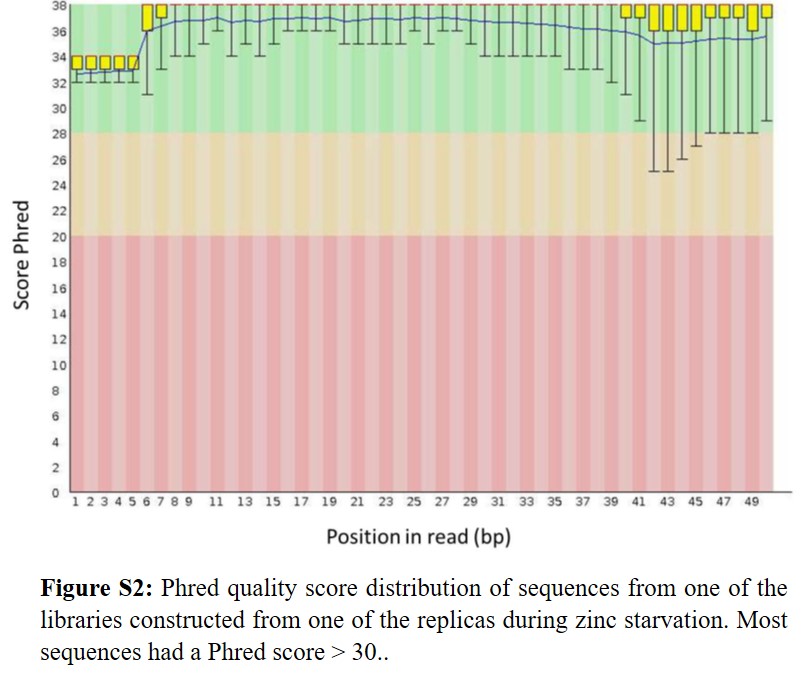

Supplement: Supplementary file 1 [file jof-09-00281-s001.zip › Figura S2.jpg]
